# The Impact of Load Carriage on Measures of Power and Agility in Tactical Occupations: A Critical Review

**DOI:** 10.3390/ijerph15010088

**Published:** 2018-01-07

**Authors:** Aaron Joseph, Amy Wiley, Robin Orr, Benjamin Schram, J. Jay Dawes

**Affiliations:** 1 Faculty of Health Sciences and Medicine, Bond University, Gold Coast, QLD 4229, Australia; aaron.joseph@student.bond.edu.au (A.J.); amy.wiley@student.bond.edu.au (A.W.); 2 Tactical Research Unit, Bond University, Gold Coast, QLD 4229, Australia; bschram@bond.edu.au; 3 Department of Health Science, University of Colorado, Colorado Springs, CO 80918, USA; jdawes@uccs.edu

**Keywords:** power, agility, mobility, tactical, police, law enforcement, military, load, personal protective equipment, body armour

## Abstract

The current literature suggests that load carriage can impact on a tactical officer’s mobility, and that survival in the field may rely on the officer’s mobility. The ability for humans to generate power and agility is critical for performance of the high-intensity movements required in the field of duty. The aims of this review were to critically examine the literature investigating the impacts of load carriage on measures of power and agility and to synthesize the findings. The authors completed a search of the literature using key search terms in four databases. After relevant studies were located using strict inclusion and exclusion criteria, the studies were critically appraised using the Downs and Black Checklist and relevant data were extracted and tabled. Fourteen studies were deemed relevant for this review, ranging in percentage quality scores from 42.85% to 71.43%. Outcome measures used in these studies to indicate levels of power and agility included short-distance sprints, vertical jumps, and agility runs, among others. Performance of both power and agility was shown to decrease when tactical load was added to the participants. This suggests that the increase in weight carried by tactical officers may put this population at risk of injury or fatality in the line of duty.

## 1. Introduction

Tactical personnel are defined as professionals whose sworn duty is to protect their community or country; that duty that can place them at risk of injury [1]. These men and women include, but are not limited to, military, fire and rescue, and law enforcement personnel [2]. Due to the nature of their occupations, these personnel may be required to perform tasks that require them to react and move very quickly, often at a moment’s notice and in life-threatening situations, such as when seeking cover when they come under enemy fire [3,4]. These personnel are also subjected to tasks that require a level of mobility; for example, the ability to negotiate obstacles like walls or fences [5,6] or perform ‘fire and maneuver tasks’ and ‘break contact’ drills [7]. ‘Fire and maneuver’ tasks and ‘break contact’ drills, for example, require personnel to perform short explosive sprints and often start and end in a lying prone position. On this basis, a degree of power and agility would be needed for tactical personnel to get to their feet from a lying prone position, sprint forward a short distance and return to a lying prone position as quickly as possible. As such, success at accomplishing tactical tasks and survival in the field is dependent, in part, on the ability of men and women who serve in tactical populations to perform tasks requiring power and agility to a high standard, or risk injury, fatality, or mission failure.

Tactical populations, by the nature of their occupations, are also required to wear and carry additional loads on a daily basis [8]. Law enforcement officers are often required to wear stab-resistant body armour, as well as other accessories on their duty belts [9]. This additional equipment can add as much as 8 kg [9] to 10 kg [10] of load to their person, with officers in specialist police units carrying as much as an additional 22 kg of load on their body [8]. Firefighters are required to carry similar loads of around 16–22 kg when on duty [11], while military personnel typically carry much heavier loads that may be in excess of 45 kg as part of their occupation [12]. The requirement to perform tasks while wearing this load may negatively impact the ability of the tactical personnel to perform their tasks effectively and safely [7,8,13]. Holewijn and Lotens [14] found that, on average, physical performance decreased by 1% per 1 kg of additional load, while Dempsey et al. [15] established that police officers decrease in performance by 13–42% while wearing ~10 kg of body armour. 

It has been well established that repeated or sustained high intensity bouts of physical activity negatively affect the ability to maintain power, speed, and agility performance among athletic populations [16,17,18]. This may not only reduce an athlete’s opportunity for success in their respective sports, but also contributes to an increased risk of injury, as neuromuscular control tends to diminish with increasing levels of fatigue [19]. Similarly, as the ‘occupational athlete’ is required to sustain prolonged activity, their ability to express force rapidly may also diminish. Furthermore, this loss of force generation ability may be exacerbated by the increased physiological burden associated with their need to carry the aforementioned loads [19,20]. For tactical personnel, any factors that reduce these physical capacities of power and agility may place personal safety and mission success at risk.

The literature suggests that load carriage can impact on tactical task performance, most notably, in this case, mobility [15]. The literature also suggests that this mobility may be relied upon by tactical personnel for survival in the field [3], especially the ability to perform the high-intensity movements described above. Furthermore, repeated high-intensity efforts can create significant muscular fatigue, which may be accentuated as load carriage demands increase [17,19]. Considering this, how load carriage might impact the discrete measures of power and agility, and as such mobility, would allow for the informing of means to mitigate the potential negative impacts associated with increased load and aid in the implementation of targeted risk mitigation strategies. On this basis, the aims of this review were to critically examine the literature investigating the impacts of load carriage on measures of power and agility in tactical occupations and to synthesize the findings.

## 2. Methods

### 2.1. Developing Search Strategy

A three-stage approach was used to identify and obtain studies that were potentially relevant to this critical review. The first stage consisted of a rapid literature review (conducted on 14 August 2017), which helped formulate the search strategy. Key search terms were identified and selected by extracting commonly used terms in the known research. Final research terms were then established by the researchers through joint collaboration. In the second stage, the aforementioned search terms were entered into the following databases: PUBMED, EMBASE, CINAHL and SPORTDiscus. These terms were modified as required to meet the individual search strategies within each database (see Table 1). Where available, the ‘humans-only’ filter was applied to rule out studies that did not include human participants. Where this option was not available as a filter, it was manually applied.

### 2.2. Inclusion and Exclusion Criteria

Once duplicates were removed, the articles were subjected to vigorous screening using carefully selected inclusion criteria. All articles were screened by title and abstract to meet the relevance of the aims of the review. Criteria for inclusion were as follows: (a) Study available in English or able to be translated into English; (b) study available in full text; (c) study used adult human participants only; (d) study involved participants carrying added load; and (e) study used a power and/or agility outcome measure.

For the purposes of this review, power was defined as the product of force on a subject and the subject’s velocity in the direction in which the force was exerted [21]. This differs from strength, as there is a speed component involved in power. Agility, on the other hand, can be defined as the skills and abilities needed to explosively change movement velocities or modes [21]. In the case for both power and agility, speed is a major component. However, accurately measuring power and agility can be difficult, and there are often disputes on the best ways to measure each. Where there was uncertainty of whether an outcome measure used in a particular study did meet the definition for power or agility, the study was reviewed, and its potential inclusion was agreed upon by consensus. After the studies were subjected to the above inclusion criteria, the remaining studies were screened using criteria for exclusion listed (Table 2).

In an effort to limit bias and accurately screen the studies derived from the literature search, two authors (A.J., A.W.) again reviewed and screened the studies separately using the criteria above. Disagreements regarding the inclusion or exclusion of any article were discussed and mediated by a third author (R.O.) before continuing the process. Through this approach, search bias, inclusion and exclusion bias, and duplication bias were limited. Finally, as part of the third stage of the search strategy, additional relevant studies, as well as grey literature, were sourced from references found in the studies retrieved from the database search and from known researchers in this field identified through the references or known to the reviewers through previous collaborations.

### 2.3. Critical Appraisal and Data Extraction

After subjecting the studies to all inclusion and exclusion criteria, the remaining studies were critically appraised using the Downs and Black checklist [22]. The checklist has 27 items designed to assess the quality for randomized control trials and non-randomized studies and outline the strengths and weaknesses of these studies and has been used in previous reviews within tactical populations [1]. The majority of the items are scored on a ‘yes’ or ‘no’ scale, awarding one point for a ‘yes’ answer and zero points for a ‘no’ answer. Item 5 on the checklist, however, is scored on a two-point scale, awarding two points for ‘yes’, one point for ‘partially’, or zero points for ‘no’ result. The final question in this checklist, which assessed statistical power of the study, is normally scored on a scale of 0–5 based on the study’s sample size. This question was modified to give one point for a ‘yes’ answer when the authors of the study reported a power analysis or zero points for a ‘no’ answer when the authors did not provide a power analysis. This modified approach to the checklist has been previously used in the literature to limit subjectivity to the question [23]. Through this approach, the maximum possible raw score became a 28, as opposed to the original maximum score of 32. 

The appraisal process described above was completed by two authors (Aaron Joseph, Amy Wiley) individually, so as to limit bias. Using the calculation of a Cohen’s kappa coefficient (k), the level of interrater agreement of the raw scores was then determined by a third author (Robin Orr). This method followed previously published guidelines that are currently used in the literature [24]. The Critical Appraisal Score (CAS) was then determined by the third author (Robin Orr) by settling any discrepancies in scores between the two raters. Following this, the scores given for each study were converted to percentages and subjected to the grading system proposed by Kennelly [25]. Kennelly’s system awards a rating based on the Downs and Black raw score given by the raters; however, the authors of this review modified the system to be presented as percentages to make it relevant to the modified Downs and Black checklist as follows: >61% as ‘good’ quality, 45–61% as ‘fair’ quality, and <45% as ‘poor’ quality. 

Once the critical appraisal of the studies was completed, pertinent data were extracted from the included studies and tabled. Information extracted included all authors, title of study, year of publication, aim of the study, participant details, and main findings that were relevant to the aims of this review.

## 3. Results

### 3.1. Study Selection and Demographics

**Figure 1:**
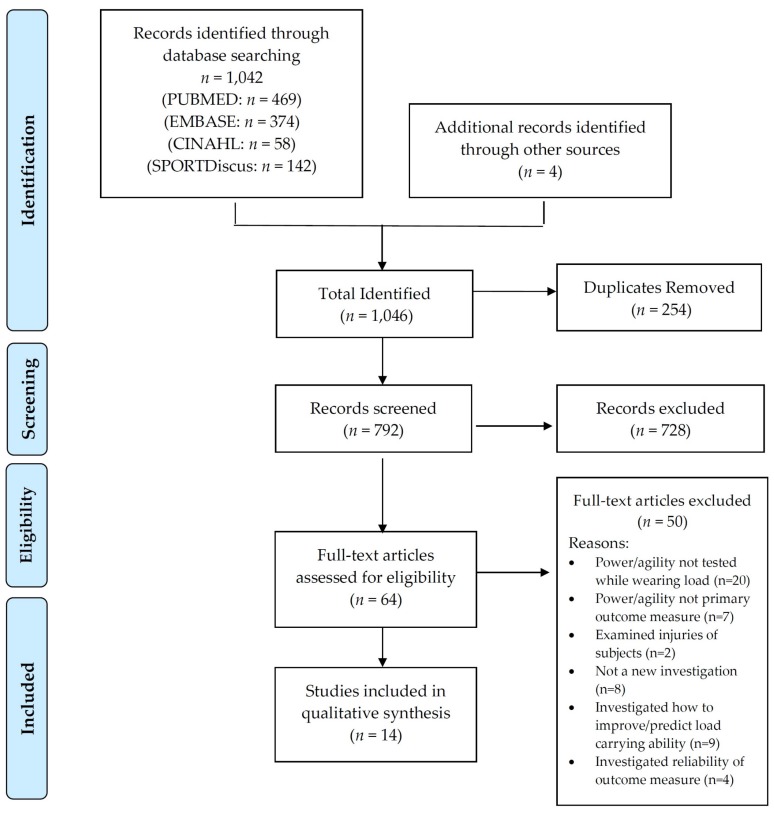
PRISMA diagram detailing the screening process of the literature review.

The PRISMA flow diagram (Figure 1) details the refinement of research articles through the critical review process. It also provides a list of the databases and search results prior to screening and removal of duplicates. In total, 1042 studies were identified across four databases, with a further four articles acquired outside the database search through other sources. Studies that used the same data set as another study were treated as duplicates and were removed. There were 254 articles removed as duplicates, resulting in 792 studies to be reviewed against the inclusion criteria. Through implementation of the inclusion criteria, 728 articles were removed, leaving 64 studies to be reviewed against the exclusion criteria. Of those studies, 50 were removed following implementation of exclusion criteria.

In total, 14 studies were deemed eligible for review and were subject to critical review. Of these studies, seven were conducted in the USA [13,26,27,28,29,30,31], four were conducted in Australia [7,8,32,33], two in New Zealand [9,15], and one in the Netherlands [14]. Nine of the studies used only male participants [8,9,13,14,15,26,27,28,33], one study used only female participants [29], three studies used both male and female participants [30,31,32], and one study did not specify gender of participants [7]. Ten of the studies [7,14,26,27,28,29,30,31,32,33] subjected military personnel to the tests and four studies [8,9,13,15] tested police officers. Two of the studies [8,9] measured the impact of load on power, five studies [13,15,27,28,32] measured the impact of load on agility, and seven studies measured both power and agility [7,14,26,29,30,31,33].

### 3.2. Critical Appraisal of Studies

The final CAS percentage scores, indicating the methodological quality of each study, are presented in Table 3, as well as information regarding the outcome measures that were used in the study and the study’s findings. The Cohen’s kappa analysis (k = 0.728) revealed an interrater agreement of ‘substantial agreement’ as per Viera and Garrett’s interpretation [24]. Four studies were graded as ‘good’ quality studies [7,8,9,15], nine were graded as ‘fair’ quality [13,14,26,27,28,29,32,33,34], and one was graded as ‘poor’ quality [31]. The mean CAS percentage for methodological quality of the included studies was 58.16%, (‘fair’ quality) with a high score of 71.43% (‘good’ quality) [7] and a low score of 42.85% (‘poor’ quality) [31].

Common weaknesses were observed in the included studies in certain areas of the Downs and Black checklist [22]. Questions dealing with external validity were often given a score of ‘0’ due to an overwhelming number of studies using only male participants, which is not representative of the whole population from which they were recruited. The facilities in which the measures were taken (for example, in fitness centers or training areas) were also not representative of the environment in which they would be performing these measures in their occupations (for example on the street, battlefield of fire ground). Questions dealing with internal validity were also often given a score of ‘0’, since most of the included studies did not make an attempt to blind the participants or assessors. This was mainly due to the nature of the studies, as it would be difficult to blind in the study given that participants would be aware of when they were or were not wearing additional load on their bodies.

### 3.3. Study Characteristics and Findings

Table 3 outlines the data extracted from the included studies, with information on the participants, specific outcome measures used, and main findings of the study. The outcome measurements for power and agility varied across the included studies. When assessing power, some studies used a sprint as the outcome measure; either a 10 m [8], 25 m [30], or 30 m [7,26,29,33] sprint with load. Other studies measured power through a loaded vertical jump test [9,14,33]. Agility was also measured through various techniques. Some studies used an agility run as the primary outcome measure [31], while others used an obstacle course or maneuverability tasks that incorporated various agility measures [7,14,15,28,29,31,33]. Agility was also measured in the form of a sprint but from a prone starting position [27,32,33]. All of the included studies used one of the measures listed above while carrying added load.

#### 3.3.1. Short Distance Sprints

Six of the included studies measured power in the form of short distance sprints. Of the studies that measured 30-m sprints [7,26,29,33], all of them showed a significant decrease in performance when additional load was added. It should be noted that in the study conducted by Pandorf et al. [29], the 30 m sprint was one leg of an obstacle course the participants had to traverse, so a slower time to complete this sprint may be due in part to fatigue from the other sections of the course or participants were conserving energy to optimize overall time to completion. In the study completed by Martin et al. [30], the 25 m sprints were conducted under five loaded conditions (1: 0.77 kg, 2: 9.41 kg, 3: 17.59 kg, 4: 29.93 kg, 5: 36.73 kg) for each participant. Each condition showed a significant decrease in performance when compared to the unloaded condition, and all loaded conditions showed a significant difference in performance from each other except for conditions 4 and 5 (29.93 kg and 36.73 kg, respectively). There was not a significant difference in the loaded (approximately 22 kg) and unloaded conditions in the 10 m sprint conducted by Carlton et al. [8], but increases in time required to complete the sprint in the loaded condition were observed. 

#### 3.3.2. Vertical Jump

Three studies included in this review measured power via a vertical jump test [9,14,33]. The studies conducted by Dempsey et al. [9] and Holewijn and Lotens [14] both showed a significant decrease in the height of vertical jump when loads of between 7.65 kg and 16 kg was added to the participant; Dempsey et al. found a decrease of 13% when loaded with 7.65 kg while Holewijn and Lotens showed a 27% loss in their loaded condition with loads of 16 kg. Taylor et al. [33] found that there were no significant decreases between each of their four tiers of loaded conditions (1: 21.6 kg, 2: 25.0 kg, 3: 26.0 kg, 4: 29.2 kg) but they did find a significant decrease in each tier when compared to the control (19.1 kg) state. Maximal effort vertical jump was only collected for descriptive purposes in the study conducted by Lewinski et al. [13], however, a 17% decrease in performance was observed while participants were wearing the 9-kg weight belt.

#### 3.3.3. Maneuverability Tasks

Numerous studies used certain maneuverability tasks to measure the performance loss in agility with added load. These tasks included traversing obstacle courses that incorporated various agility measures [14,29,33], fire and movement simulations [7,33], and agility drills [15,28,31]. Obstacle course times across the studies showed significant decreases in completion times when load was added. However, in the study conducted by Taylor et al. [33], obstacle course times were only significantly slower in weight tiers two-four (2: 25.0 kg, 3: 26.0 kg, 4: 29.2 kg) when compared to the control state (19.1 kg). Holewijn and Lotens [14] divided their obstacle course into three segments (A, B, C), and while only obstacle course A showed a significant decrease on its own, overall the combination of the three courses showed a significant decrease in completion time when participants were wearing loads of 16 kg. Fire and movement simulations all showed significant decreases in time as well. Agility drills, such as the modified MANUF test and acceleration tasks simulating exiting a vehicle, often showed loss of agility performance; however one study [31] did not observe this result. DeMaio et al. [31] used a box drill that incorporated sprinting forward, side shuffling, and running backwards four times around a 10 × 10 m box, but this box agility test was not significantly affected by personal protective equipment (PPE) (9.8 ± 0.9 kg).

#### 3.3.4. Prone-Start Sprint

Sprints from a prone starting position require a considerable amount of agility. This outcome measure was used to observe the performance of agility under load as opposed to power. Three studies used this measure [27,32,33], and these studies unanimously observed significant effects of load on the agility of the participant. The distance of the sprints varied from 5 m [33] to 30 m [27,32], showing that agility is affected over a variety of distances, with loads ranging from 12.1 to 30.4 kg.

#### 3.3.5. Agility Run

Two of the included studies measured agility through the use of an agility or shuttle run [30,31]. Both studies observed that the time to complete increased when load was added to a significant standard. Martin [30] states that with respect to load, significant differences were found between the performance for all loads for the men and for all loads (1: 0.77 kg, 2: 9.41 kg, 3: 17.59 kg, 4: 29.93 kg, 5: 36.73 kg) except load conditions 4 and 5 (29.29 and 36.09 kg respectively) for the women.

## 4. Discussion

This critical review aimed to identify and critically appraise the methodological quality of studies investigating impacts of load carriage on measures of power and agility and to synthesize their findings. Four main areas of discussion were formed based off the results gathered: (1) the quality of the included studies; (2) the impact of added load on outcome measures of power; (3) the impact of added load on outcome measures of agility; and (4) implications of these findings to tactical population based on the volume of evidence reviewed and potential recommendations to mitigate these implications.

### 4.1. Quality of Research

The methodological quality of the included studies as a whole was deemed ‘moderate’ based on the grading system by Kennelly [25], with the mean CAS percentage at 58.16%. While this score is not considerably high, it should be noted that the mean score was largely influenced by the majority of the included studies being marked lower in certain areas of the Downs and Black checklist [22] dealing with blinding the participants and assessors (Questions 14, 15, 23). Due to the nature of these studies, it is very difficult to blind the participants, as the participants were either carrying additional load or not. As such, these questions typically scored a ‘0’, causing a considerable reduction in the overall score. Similarly, Question 13 on the checklist, which relates to the environment in which the participants were tested being representative of the environment in which they normally work in, had the majority of studies score a ‘0’ due to the difficulty of replicating these measures in an operational tactical environment. This disparity is highlighted when heightened senses and emotions of the officer under enemy fire are taken into account; a feat difficult to truly replicate when taking the safety of the officer into consideration.

Bearing these considerations in mind, it should be noted that the quality of these studies was acceptable overall. Furthermore, based on the volume of research available, the findings of added load carriage on measures of power and agility can be considered with confidence.

### 4.2. The Impact of Load Carriage on Power

Power was shown to decrease across the included studies when additional tactical load was added to the participant. Both short-distance sprints and vertical jump tests were shown to have significantly lower results in the loaded conditions in the majority of the studies. Carlton et al. [8] did not find a significant difference when measuring added load carriage over a 10 m sprint, but this may be largely due to the small sample size of their study (*n* = 6). This study did show an increase in time to complete the 10-m sprint when the 22.8 kg (±1.8 kg) load was added to the members of the specialist police unit, and it can be inferred that if more officers participated in this study, the results may have shown statistically significant decreases in performance. Overall, additional load was shown to have the most effect on short distance sprints in respect to completion time. This indicates that when under heavy load, the tactical officer will typically require a significantly greater amount of time to reach his or her destination safely. 

The ability to generate power is necessary for explosive movements that the tactical officer performs in the field, such as sprinting to seek cover or jumping to negotiate a high fence. Increases in tactical load may come at the expense of the officer’s ability to successfully perform these movements quickly, and could mean risking the officer’s safety or the success of the mission. While this time period (i.e., seconds) may be considered very small it must be considered in context. For example, the cyclic rate of an AK 47 automatic assault rifle is around 600 rounds per minute and, on this basis, covering a distance one second slower could leave tactical personnel exposed to an additional 10 rounds while seeking cover from an armed offender or enemy combatant utilizing one of these weapons. This information should be taken into consideration when sending an officer into a situation in which explosive maneuvers may be required to survive.

### 4.3. The Impact of Load Carriage on Agility

As was the case with power, agility was shown to suffer when the tactical officer was subjected to additional load. As a whole, the performance of all of the outcome measures used to observe agility decreased as load carriage increased. This was observed as many studies used tiers of weight in their experiments, and the heavier tiers typically showed increasingly significant differences from the lighter tiers. DeMaio et al. [31] observed that their box agility drill was not significantly affected under load, however, this may be due in part to the relatively low weight of the PPE worn during the experiment (9.8 ± 0.9 kg). Incidentally, time to complete the drill did increase.

It should be taken into account, however, that while increases in times to complete obstacle courses were observed across the included studies, this may be due in part to the amount of space that the increased load occupied. For example, the increase in completion times for crawling underneath wires in the study by Holewijn and Lotens [14] could have been due to the fact that there was reduced space for the officer to crawl through due to the large backpack they carried. 

Apart from the agility to traverse or circumvent obstacles rapidly, which may give an opponent an advantage, it should be noted that reduction in agility may increase the tactical officer’s risk of a slip, trip, or fall. Research by Park et al. [35] identified that firefighter foot clearance when stepping over a 30 cm hurdle decreased and contacts with the hurdle increases with they were loaded (9.1 kg). This finding is of note given that slips, trips, and falls are a leading mechanism of injury in tactical personnel [36].

### 4.4. Implications and Recommendations

These results are especially pertinent for the tactical population. Load carriage represented by the weight of additional equipment carried by tactical personnel significantly decreased their power and agility, and therefore their mobility. Although the equipment and armour that tactical populations are required to carry may offer additional protection or necessary supplies to the person, the load may reach a point to where mobility is suffering [13]. This decrease in mobility is also directly correlated with an increase in exposure to enemy fire in the field [3]. This information suggests that these personnel are placed at a much higher risk for injury or fatality if the loads are such that they reach a point where the detrimental effects of load carriage on mobility are greater than the potential protection they provide.

Considering this, it is critical that training procedures and policies for tactical personnel include physical conditioning to specifically increase and optimize the carrier’s ability to generate power and move with agility [3,13]. It is also recommended that, where possible, loads carried be reduced as much as possible prior to any tasks that require power and/or agility [15]. 

### 4.5. Limitations

Key limitations of this review identified included a potential language bias and restriction to the majority of research to male participants. Given that only English databases were searched, in conjunction with English search terms, the potential for a language bias is present. Furthermore, while the quality of the studies was of good standard, many studies only used young male participants in their research. Considering that female personnel serve in tactical populations and perform the same operational tasks as male personnel, there was very little research into the effects of load carriage on female performance of power and agility. Given the relationship between fat mass and the ability to generate power [4] and that, in general, females have a higher fat mass than males, female personnel may be more adversely impacted by loads than male personnel. As such, more research is required to understand the impact load may have on the mobility of female personnel to identify whether any differences exist due to the sex of the load carrier. Finally, it should be noted that the majority of this research was conducted in military populations, with a limited number of studies in law enforcement and no studies in firefighter populations. Considering this, with all these tactical populations required to carry loads, it is anticipated that the impacts of load carriage on measures of power and agility identified in this study will transcend to all tactical personnel.

## 5. Conclusions

In conclusion, this review observed that added load may have significant impacts on the ability of tactical personnel to perform activities that require power and agility. This may in turn reduce their mobility and increase their risk of injury and potentially mortality and operational success. On this basis, measures that optimize the ability of tactical personnel to generate power and agility while carrying load, such as physical conditioning and load reduction, is of importance. More research is required to take measures to reduce the weight carried by tactical personnel without compromising the safety or utility the load may offer.

## Figures and Tables

**Table 1:** Databases and search terms used during literature search.

Database	Search Terms
PUBMED (24 August 17)	((“Military Personnel”[Mesh]) OR (“Law Enforcement”[Mesh]) OR (“Police”[Mesh]) OR (“Firefighters”[Mesh]) OR tactical OR military OR police OR firefight* OR “law enforcement”) AND (load* OR “body armor” OR “body armour” OR equipment) AND (power OR agility OR mobility OR sprint OR jump OR obstacle)
EMBASE (24 August 17)	(Load* OR Equipment OR “body armor” OR “body armour”) AND (Power OR Sprint OR “vertical jump” OR jump OR Agility OR “Obstacle course” OR mobility) AND (Tactical OR Military OR “military personnel” OR police OR officer* OR firefighter* OR “law enforcement” OR soldier* OR army OR navy)
CINAHL (24 August 17)	(Load* OR Equipment OR “body armor” OR “body armour”) AND (Power OR Sprint OR “vertical jump” OR jump OR Agility OR “Obstacle course” OR mobility) AND (Tactical OR Military OR “military personnel” OR police OR officer* OR firefighter* OR “law enforcement” OR soldier* OR army OR navy)
SPORTDiscus (24 August 17)	(Load* OR Equipment OR “body armor” OR “body armour”) AND (Power OR Sprint OR “vertical jump” OR jump OR Agility OR “Obstacle course” OR mobility) AND (Tactical OR Military OR “military personnel” OR police OR officer* OR firefighter* OR “law enforcement” OR soldier* OR army OR navy)

* is part of the search terms and symbols. There is no actual meaning it just tells the search engine to look for any versions of that word.

**Table 2:** Exclusion criteria and examples of excluded studies.

Exclusion Criteria	Example
Study was not a new investigation	Study was a critical or systematic review
Study examined injuries of participants	Study predicted injury rate of participants by completing a power or agility outcome measure
Neither power nor agility were the primary outcome measure used in the study	Study examined effects of load on physiological responses such as VO2max or heart rate while utilizing a power or agility outcome measure
Participants were not wearing added load during the measurement of power or agility in the study	Study examined the effect of training with load on improvements of power and/or agility without load
Study investigated how to improve or predict load carrying ability	Study reported on how to improve load carrying ability through resistance or aerobic training
Investigations of reliability of outcome measures	Study investigated the reliability of a power or agility outcome measure through load bearing techniques

**Table 3:** Participants, loading conditions, outcome measures, and main findings.

Authors (Year) and Title	Participant Details	Loading Conditions	Outcome Measures Used	Main Findings	Critical Appraisal Score (%)
Carlton et al. (2014) [8] The impact of occupational load carriage of the mobility of the tactical police officer	Active officers of a police Tactical Operations Unit (*n* = 6) Male (100%) Mean Age (SD): 33.3 ± 4.13-yrs Mean Height (SD): 177.0 ± 11.8-cm Mean Weight (SD): 89.2 ± 13.2-kg Mean years of experience in the police force (SD): 10.9 ± 5.1-yrs	Unloaded Body armour, helmet, primary (M4) and secondary (9 mm Glock) weapons (22.8 ± 1.8-kg)	10-m in line sprint 25-m simulated patrol Two 10-m dummy (70-kg) drags	No significant difference in 10-m Sprint (*p* = 0.91) or 25-m Tactical Movement (*p* = 0.146) times between UL and TL conditions, mean increases in time were noted There was a significant increase in mean time to complete the 10-m dummy drag during the TL condition compared to the UL condition (*p* = 0.009).	67.85%
DeMaio et al. (2009) [31] Physical performance decrements in military personnel wearing personal protective equipment	Physically active volunteers from various U.S. Military Commands (*n* = 21) Males (90.5%) Females (9.5%) Males: Mean age: 24.2 ± 3.7-yrs Mean height: 180.1 ± 5.4-cm Mean weight: 82.9 ± 11.0 kg Females: Mean age: 23.0 ± 0-yr Mean height: 161.3 ± 14.4-cm Mean weight: 56.1 ± 6.7-kg	Military issued battle dress uniforms + PPE system: 9.8 ± 0.9 kg	Shuttle Run 300-yd (274-m): 25-yd (23-m) repeats Box Drill 4 × 10-yd (9.1-m): sprint forward, side shuffle, run backwards, carioca Upper Extremity Power: rope pull and dummy drag	Shuttle Run was significantly affected by added PPE (*p* < 0.001) Box Agility Test was not significantly affected by added PPE (*p* = 0.28) Rope Pull and Dummy Drag was not significantly affected by added PPE (*p* = 0.42)	42.85%
Dempsey et al. (2013) [15] Impact of police body armour and equipment on mobility	New Zealand Southern Region District Police force (*n* = 52) Male (100%) Mean Age: 37 ± 9.16-yrs Mean Height: 180.68 ± 6.12-cm Mean Weight: 90.21 ± 11.59-kg Mean BMI: 27.61 ± 3.09	Unloaded Fitted stab-resistant body armour and simulated duty gear (7.65 ± 0.73-kg)	Acceleration Task—to simulate exiting a vehicle Maneuverability Task	Acceleration Task was significantly effected in loaded condition (+0.2-s, *p* < 0.001). Maneuverability Task was significantly effected in loaded condition (+2.1-s, *p* < 0.001).	64.28%
Dempsey et al. (2014) [9] Body armour; the effect of load, exercise and distraction on landing forces	New Zealand Southern Region District Police force (*n* = 52) Male (100%) Mean Age: 37 ± 9.16-yrs Mean Height: 180.68 ± 6.12-cm Mean Weight: 90.21 ± 11.59-kg Mean BMI: 27.61 ± 3.09	Unloaded Fitted stab-resistant body armour and simulated duty gear (7.65 ± 0.73 kg)	Vertical Jump Height	There was a significant reduction in jump height when loaded (*p* < 0.001).	64.28%
Hasselquist et al. (2008) [26] Understanding the physiological, biomechanical, and performance effects of body armor use	U.S. Army military personnel (*n* = 11) Mean Age: 20-yrs Mean Height: 1.8-m Mean Weight: 79.7-kg	Interceptor Body Armor tactical vest (including collar, groin protector, protective inserts) = 8.7 kg, 0.411 m^2^ coverage Three types of extremity armour: EXT 1: 5.6 kg, 0.717 m^2^ coverage EXT 2: 6.4 kg, 0.775 m^2^ coverage EXT 3: 5.6 kg, 0.926 m^2^ coverage	5 × 30-m Rushes Obstacle Course Runs: a set of four plastic hurdles, 0.6 m high; a field of 9 rubber cones delineating a zig zag running pattern, 27 m long and 1.5 m wide; a crawl space of wood/wire, 0.6 m high, 0.9 m wide, 3.7 m long; a horizontal shimmy pipe, 3.7 m long; a 1.4 m high sheer wooden wall with footholds or ropes; a 27 m straight run; a jump and reach activity; stair climbing	Rush and obstacle course performance scores were significantly poorer with the armour vest than without the armour vest. *	57.14%
Holewijn and Lotens (1993) [14] The influence of backpack design on physical performance	Royal Netherlands Army infantry soldiers (*n* = 10) Male (100%) Mean Weight: 75-kg (67–85-kg) Mean Height: 182-cm (175–187-cm)	Unloaded 16 kg Carrying Harness: Weight low on back Weight high on back Weight distributed	Vertical Jump Obstacle Course A (crawling underneath wires for 10-m) Obstacle Course B (stepping stones, climbing bars, oblique wall) Obstacle Course C (steeples, climbing on a platform) Mobility Task: running 3 × 8-shaped laps around 2 uprights placed 2-m apart, while ducking under a crossbar (1.2-m)	Average loss of performance due to carriage of a mass of 16-kg: Vertical Jump: 27% loss Obstacle Course: 31% loss Mobility Task: 11% loss	53.57%
Hunt et al. (2016) [7] Tactical combat movement: inter-individual variation in performance due to the effects of load carriage	Royal Australian Air Force Airfield Defence Guards (*n* = 14) Mean age: 21.7 ± 2.4-yrs Mean height: 181.4 ± 8.0-cm Mean body mass: 81.0 ± 9.0-kg Mean fat mass: 10.5 ± 3.5-kg Mean skeletal muscle mass: 40.6 ± 4.8-kg Mean shuttle run-predicted maximal aerobic power: 49.6 ± 2.9-mL kg^−1^ min^−1^	Weapon: 4.7-kg Chest Webbing: 2.2–2.4-kg plus 2.8, 7.7, or 12.5-kg of load Body Armour: 8.6–12-kg Helmet: 1.2–1.4-kg Break Contact Total Load (kg): A: 9.8 ± 0.5 B: 14.6 ± 0.2 C: 20.8 ± 1.1 D: 25.5 ± 0.8 E: 30.3 ± 0.9 Fire and Movement Total Load (kg): A: 9.8 ± 0.3 B: 14.6 ± 0.3 C: 20.9 ± 0.9 D: 25.6 ± 1.0 E: 30.1 ± 0.9	Break Contact Simulation: 5 × 30-m sprints, 44-s cycle Fire and Movement Simulation: 16 × 6-m bounds, 20-s cycle controlled by a digital audio cadence, starting in prone firing position, finishing in kneeling firing position	Break Contact Simulation: sprint duration increased by 0.8%/kg increase in external load. Increase in load lead to a significant increase in sprint duration and a sig decrease in peak velocity (*p* < 0.001). Fire and Movement Simulation: bound duration increased by 1.1%/kg increase in external load for the re and movement simulation.	71.43%
Jaworski et al. (2015) [28] Changes in combat task performance under increasing loads in active duty marines	U.S. Marine Corps Infantry Battalions active duty military personnel (*n* = 18) Male (100%) Mean Age: 21 ± 2.5-yrs Mean Weight: 82 ± 10.1-kg Mean BMI: 25.6 ± 1.2-km/m^2^ Mean HR Average: 175 ± 2.8-bpm Mean Max HR: 187 ± 2.7-bpm Average months of active duty service: 33.4 ± 20-mo	4 Load Conditions: 1 (Neat): utilities and boots 2 (15% BW ± 2-lb): utilities and boots, body armour, helmet 3 (30% BW ± 2-lb): utilities and boots, body armour, helmet, hydration system, pack loaded with sand 4 (45% BW): utilities and boots, body armour, helmet, pack loaded with hydration system and sand	Modified MANUF portion: Split 1: 25-yd sprint, J-hook turn, 25-yd low crawl-high crawl Split 2: 75-yd casualty carry (10-yd drag, 65-yd fireman carry) Split 3: ammo can run to grenade toss (75-yd) Split 4: Ammo can run to end (75-yd) Split 5: MANUF end to ISMT marksmanship trainer	MANUF total time significantly increased with increasing load (*p* < 0.0001). There was a significant effect of split times (except split 2). There was a significant relationship between total time to completion and total load (*p* < 0.0001).	53.57%
Lewinski et al. (2015) [13] The influence of officer equipment and protection on short sprinting performance	Law Enforcement Students (*n* = 20) Male (100%) Mean age: 21.25 ± 2.61-yrs Mean mass: 80.74 ± 11.79-kg Mean BF%: 11.98 ± 5.87% Mean vertical jump: 0.54 ± 0.10-m Mean VO2 Max: 53.02 ± 4.72-mL^−1^ kg^−1^ min^−1^	Training uniform and boots 9.07-kg weight belt (11.47 ± 1.64% of participants’ body mass)	8 × max effort 9.1-m sprints, 1-min recovery (6 complete strides), 4 different starting positions: forwards, backwards, 90° turn to the left, 90° turn to the right	Overall performance decrease of 5% for stride velocity Stride length unaffected 17% decrease in VJ performance	57.14%
Loverro et al. (2015) [27] Use of body armor protection with fighting load impacts soldier performance and kinematics	Soldiers (*n* = 13) Male (100%) Mean Age: 21.2 ± 2.5-yrs Mean Height: 1.8 ± 0.6-m Mean Weight: 83.4 ± 9.8-kg	IOTV Light: IOTV with soft armour only (4.8-kg) No Fighting Load = 12.1-kg With Fighting Load = 23.1-kg PC Heavy: PC with front, back, and side plates (9.8-kg) No Fighting Load = 17.1-kg With Fighting Load = 28.1-kg IOTV Heavy: IOTV with front, back, and side plates (12.2-kg) No Fighting Load = 19.4-kg With Fighting Load = 30.4-kg	Rush Task: 10 × 30-m rushes, with 9 drop to prone and turns performed successfully	There was a significant effect of body armour on individual (*p* = 0.037) and total rush time (*p* = 0.017) during the rush task. IOTV Heavy individual rush time was significantly longer compared to IOTV light (*p* = 0.004). There was no significant difference noted between IOTV Heavy and PC Heavy individual rush times (*p* = 0.05).	60.71%
Martin et al. (1985) [30] The effect of carried loads on the combative movement performance of men and women	Penn State Army R.O.T.C students (*n* = 30) Men (53.3%) Women (46.7%) Mean Age (males): 20.9 ± 1.5-yrs Mean Age (females): 20.7 ± 1.5-yrs Mean Height (males): 175.1 ± 7.1-cm Mean Height (females): 165.9 ± 5.4-cm Mean Body Mass (males): 69.8 ± 7.2-kg Mean Body Mass (females): 59.9 ± 9.3-kg	Men (*n* = 16) Absolute Mean Values for Load Conditions: 1: 0.77-kg 2: 9.41-kg 3: 17.59-kg 4: 29.93-kg 5: 36.73-kg Women (*n* = 14) Absolute Mean Values for Load Conditions: 1: 0.59-kg 2: 9.07-kg 3: 16.95-kg 4: 29.29-kg 5: 36.09-kg	25-yd Sprint Simple Agility Run Standing Long Jump	There was a 12.50%, 19.39%, 32.14%, 34.44% increase in general 25-yd sprint times for loaded conditions 2, 3, 4, 5, respectively, in comparison to loaded condition 1. There was a 7.34%, 13.30%, 27.01%, 26.18% increase in general agility run times for loaded conditions 2, 3, 4, 5, respectively, in comparison to loaded condition 1. There was a 13.42%, 18.18%, 28.57%, 31.60% decrease in general standing long jump distances for loaded conditions 2, 3, 4, 5, respectively, in comparison to loaded condition 1. *p*-values were not reported.	53.57%
Pandorf et al. (2002) [29] Correlates of load carriage and obstacle course performance among women	Female Soldiers (*n* = 11) Women (100%) Mean Body Mass: 61.3 ± 6.7-kg (52.5–72.0-kg) Mean Height: 166.0 ± 6.5-cm (154.7–174.8-cm) Mean Age: 25.3 ± 5.5-yr (19.4–38.2-yr) Body Fat %: 25.7 ± 3.22% (20.6–31.5%) APFT Score: 256 ± 24 (216–290)	Fighting load: battle dress uniform, boots, body armour, Kelvar helmet, equipment belt, load-carriage vest, dummy grenades, ammunition clips, M-16 rifle, 14.2 ± 0.59-kg Approach load: fighting load + 13.6-kg in a backpack, 27.2 ± 1.2-kg	Hurdles: 4 × 46-cm high plastic hurdles, 2.1-m apart Zigzag Course: zigzag pattern through 9 × plastic cones, adjacent cones 1.5-m apart laterally, 3.4-m apart along a 26.8-m course Straight Sprint: 28.7-m straight sprint	There was a 25.93%, 11.76%, 17.05% decrease in performance for the hurdles, zigzag, and straight sprint, respectively, between fighting load and approach load. *	60.71%
Taylor et al. (2016) [33] Balancing ballistic protection against physiological strain: evidence from laboratory and field tests	Active service soldiers (*n* = 31) Males (100%) Mean age (SD): 23.0-yrs (4.0) Mean body mass (SD): 84.7-kg (13.4) Mean height (SD): 1.79-m (0.07)	Control (tier-zero): 19.1-kg Tier-one armour: 21.6-kg Tier-two armour: 25.0-kg Tier-three armour: 26.0-kg Tier-four armour: 29.2-kg	Fire and Movement Simulation: 12 × 5-m sprints starting from prone firing position, 25-s duty cycle (N = 17) Obstacle-Avoidance Test: 5 evenly spaced poles over 40-m (N = 25) Combat-Rush Simulation: 30-m straight-line sprint from standing start (N = 27) Vertical-Jump Test (N = 22)	Time to complete the Fire and Movement Simulation was significantly slower in tiers-two, -three, -four than control, with tier-four also being sig. slower than tier-one (*p* < 0.05). There was no significant difference in time to complete the Obstacle Avoidance Test between the control and tier-one (*p* > 0.05), times for tiers-two, -three, and -four were sig slower than the control state (*p* < 0.05). Time for the Combat-Rush Simulation was sig. slower for the tier-four state compared to the control and tier-one states (*p* > 0.05). There were no sig differences in Vertical Jump among tiers-one through to four (*p* > 0.05), tiers-two, -three, -four were sig. less than the control state (*p* < 0.05).	50.00%
Treloar et al. (2011) [32] Effect of load carriage on performance of an explosive, anaerobic military task	Soldiers (occupational specialties) (*n* = 17) Male (70.5%) Female (29.5%) Mean age: 23.1 (18–40-yrs) Mean mass: 78.2 ± 13.0-kg Mean height: 178.6 ± 7.1-cm Yrs experience: 0.25–18-yrs	Unloaded Combat uniform + 21.6-kg (webbing, weapon, CBA, helmet)	5 × 30-m sprints at 44-s intervals—starting in the prone position	Additional load significantly increased mean 30-m sprint performance time by 31.5% (*p* < 0.01).	53.57%

* *p*-value was not reported.
